# Multiple Interactions Between Cancer Cells and the Tumor Microenvironment Modulate TRAIL Signaling: Implications for TRAIL Receptor Targeted Therapy

**DOI:** 10.3389/fimmu.2019.01530

**Published:** 2019-07-03

**Authors:** Margot de Looff, Steven de Jong, Frank A. E. Kruyt

**Affiliations:** Department of Medical Oncology, University Medical Center Groningen, University of Groningen, Groningen, Netherlands

**Keywords:** TRAIL, TME, cytokines, cancer, immune suppression

## Abstract

Tumor necrosis factor (TNF) related apoptosis-inducing ligand (TRAIL) signaling is far more complex than initially anticipated and can lead to either anti- or protumorigenic effects, hampering the successful clinical use of therapeutic TRAIL receptor agonists. Cell autonomous resistance mechanisms have been identified in addition to paracrine factors that can modulate apoptosis sensitivity. The tumor microenvironment (TME), consisting of cellular and non-cellular components, is a source for multiple signals that are able to modulate TRAIL signaling in tumor and stromal cells. Particularly immune effector cells, also part of the TME, employ the TRAIL/TRAIL-R system whereby cell surface expressed TRAIL can activate apoptosis via TRAIL receptors on tumor cells, which is part of tumor immune surveillance. In this review we aim to dissect the impact of the TME on signaling induced by endogenous and exogenous/therapeutic TRAIL, thereby distinguishing different components of the TME such as immune effector cells, neutrophils, macrophages, and non-hematopoietic stromal cells. In addition, also non-cellular biochemical and biophysical properties of the TME are considered including mechanical stress, acidity, hypoxia, and glucose deprivation. Available literature thus far indicates that tumor-TME interactions are complex and often bidirectional leading to tumor-enhancing or tumor-reducing effects in a tumor model- and tumor type-dependent fashion. Multiple signals originating from different components of the TME simultaneously affect TRAIL receptor signaling. We conclude that in order to unleash the full clinical potential of TRAIL receptor agonists it will be necessary to increase our understanding of the contribution of different TME components on outcome of therapeutic TRAIL receptor activation in order to identify the most critical mechanism responsible for resistance, allowing the design of effective combination treatments.

## Introduction

TRAIL receptors (TRAIL-Rs) are able to selectively induce apoptosis in cancer cells and are considered promising therapeutic targets. However, in clinical studies the efficacy of TRAIL-R agonists has been rather disappointing thus far. Novel formulations of TRAIL-R agonists able to more efficiently cluster and activate TRAIL-Rs have been developed and may lead to better therapeutic response (1). However, the fact that TRAIL signaling is more complex than initially thought hampers the successful use of these receptor agonists. For example, TRAIL signaling was found to have protumorigenic effects in apoptosis resistant tumor cells leading to unwanted stimulation of proliferation and metastatic spread (2, 3). In addition to cell autonomous mechanisms responsible for TRAIL resistance and non-canonical signaling also cell extrinsic signals have been identified that modulate the TRAIL pathway.

Tumors resemble organs as they contain heterogeneous (tumor) cell populations with distinct differentiation status and cellular functions such as blood vessels, immune cells, and fibroblasts. The non-cancer cell compartment of a tumor is known as the TME and has been irrefutably demonstrated to play a key role in tumorigenesis, tumor progression, and therapeutic efficacy (4–6). Importantly, dynamic changes in the TME accompany tumor progression and therapeutic resistance and strategies aimed at reprogramming the cellular TME toward an antitumor state provide a promising therapeutic approach.

In this review, we focus on the impact of the TME on the outcome of TRAIL signaling in tumor cells. This includes endogenous TRAIL/TRAIL-R signaling being part of immune effector cell functioning and tumor immune surveillance as well as modulation of therapeutic efficacy of TRAIL-R agonists by bidirectional tumor/stroma cell signaling and specific biochemical and biophysical properties characteristic of the TME. The nature and implications of these pleiotropic interactions are highlighted and consequences for the efficacy of TRAIL-based therapy are discussed.

## TRAIL Receptor Targeted Cancer Therapy

TRAIL receptor agonists have been developed to induce apoptosis selectively in tumor cells, while preserving normal cells (7–9). Depending on their binding characteristics these agonists bind to both, or either one of the apoptosis-inducing receptors TRAIL-R1 and -R2. These receptors share a death effector domain required for ligand-induced formation of the death-inducing signaling complex (DISC) consisting of FAS-associated death domain (FADD) and procaspase-8, leading to caspase-8 activation and subsequent caspases-dependent apoptosis. This so called extrinsic apoptotic pathway is connected to the intrinsic or mitochondrial apoptotic pathway via the BCL2-family member BID. Caspase-8-dependent BID cleavage produces truncated (t)BID that via interactions with proapoptotic BAX and BAK disrupt mitochondrial membranes resulting in the release of proapoptotic factors such as cytochrome C, a co-factor for apoptosome formation resulting in activation of initiator procaspase-9 (10). TRAIL-R agonists are usually designed to have reduced binding affinity for decoy TRAIL-Rs, named TRAIL-R3 and TRAIL-R4. These membrane receptors have a TRAIL-binding domain but lack cytoplasmic domains required for apoptosis activation, whereas a fifth TRAIL binding protein named osteoprotegerin (OPG) is soluble and also able to sequester TRAIL, thus suppressing TRAIL-R1/R2-dependent apoptosis (11).

In spite of the anticipated powerful therapeutic potential of TRAIL-R agonists, apoptosis resistance is often encountered in cell culture and *in vivo* cancer models, providing an explanation for disappointing results in clinical studies (12–14). Importantly, TRAIL-Rs were found to induce non-canonical signaling involving activation of pro-inflammatory, pro-survival, and proliferation pathways leading to protumorigenic and even metastasis-promoting effects (2, 3). Non-canonical signaling is predominantly mediated by TRAIL-R1 and -R2 and involves the formation of a secondary signaling complex consisting of among others, receptor-interacting serine/threonine protein kinase 1(RIPK1), Tumor necrosis factor (TNF) receptor associated factor 2 (TRAF2) and TNF receptor associated death domain (TRADD) (2, 15). Subsequently, this signaling complex is able to activate various protumorigenic pathways including IκB/NF-κB, MAPK/ERK, STAT3, PI3K, Akt, JAK2, and Src.

TRAIL resistance has been often regarded as a tumor-autonomous property and various apoptosis resistance mechanisms have been identified such as absence of caspase-8 or elevated expression of various apoptosis blocking proteins including cellular FLICE-like inhibitory protein (cFLIP), X-linked inhibitor of apoptosis proteins (XIAPs), antiapoptotic BCL-2 family members, which have been extensively reviewed elsewhere (11, 16, 17). However, cell extrinsic signals derived from the TME can also modulate TRAIL apoptotic signaling. Current evidence for such interactions and consequences for therapy are discussed below.

## The Tumor Microenvironment

The TME consists of cellular components including various myeloid and lymphoid cells, fibroblasts and endothelial cells that via direct interactions or biochemical cues (auto-, para-, and endocrine signaling) communicate with tumor cells. In addition, a non-cellular TME can be distinguished consisting of extracellular matrix (ECM), mechanical pressure and tumorigenic conditions like acidity, hypoglycemia and hypoxia that impact tumor behavior (18). The fate of a tumor is dependent on dynamic properties of the TME ranging from anti- to protumorigenic. The antitumorigenic TME encompasses normal fibroblasts (NF), dendritic cells (DCs), natural killer (NK) cells, cytotoxic T cells, and M1-activated tumor-associated macrophages (TAMs) involving the activity of proinflammatory cytokines. The protumorigenic TME, on the other hand, is associated with immune suppressive effects of M2-activated TAMs involving production of anti-inflammatory cytokines, myeloid-derived suppressor cells (MDSC), regulatory T (Treg) cells and B cells, cancer-associated fibroblasts (CAFs) producing aberrant ECM, and TIE2-expressing monocytes and mast cells with angiogenesis stimulatory activity. Similar to TAMs, neutrophils and T helper (Th) cells can have both pro- and antitumorigenic activity depending on tumor and immune context. For a comprehensive review of the cellular TME and impact on tumor progression and tumor cell dissemination see Quail and Joyce (18).

## Regulation of Endogenous TRAIL by the TME

### Physiological Role of TRAIL

TRAIL has been identified as a key mediator of the innate immune response including a role in tumor immune surveillance. Endogenous TRAIL, encoded by the *TNFSF10* gene, is a 281 amino acid (aa), 33 kDa type II transmembrane protein with a small intracellular domain of 17 aa (8, 19). The extracellular domain of TRAIL can be cleaved by cysteine proteases to produce soluble TRAIL (~20 kD). TRAIL and TRAIL-Rs are expressed in various tissues including immunogenic organs like spleen and thymus. Indeed, a variety of innate and adaptive immune cells express TRAIL such as monocytes, macrophages, DCs, NK cells, and cytotoxic T cells (CTLs) (20, 21). TRAIL and TRAIL-R expression is regulated by a variety of factors depending on the cellular context. For example, IFNs can activate transcription of *TRAIL* via the IRF1/STAT3 complex. Furthermore, *TRAIL* and *TRAIL-R* transcription is regulated by stress-induced factors like nuclear factor of activated T-cells (NFAT), Forkhead Box (FOX) proteins, NF-κβ, C/EBP homologous protein, activator protein 1 (AP1), and p53 in both immune and transformed cells (22, 23).TRAIL signaling can regulate adaptive immune cells by removing aberrantly activated T effector cells maintaining T cell homeostasis. For example CD8+ T cell memory expansion is regulated by CD4+ T helper (Th1) cells via TRAIL dependent apoptosis (24).

Besides being a cytotoxic effector of immune cells in infectious diseases, TRAIL expressing immune cells also play a role in tumor suppression, although not in a consistent way (20, 25). For example, in mice having only one Trail-R, *Trail-R* knockout had no effect on incidence of spontaneous tumor development in siblings obtained from hybrid *APC–/–* (intestinal adenomas) or *p53–/–* (lymphomas) mice (26), whereas siblings of *Trail–/–* and *P53*+*/–* mice developed more sarcomas and lymphomas (27). Further, monitoring carcinogen-induced tumorigenesis in *Trail–/–* mice vs. controls demonstrated a tumor suppressive effect of Trail (28). Intriguingly, in this study no differences were detected on primary tumor formation, however, Trail-R deficient mice showed enhanced metastatic spread to lymph nodes suggesting particularly Trail-mediated suppressive effects on disseminating tumor cells (29). Thus, the TRAIL/TRAIL-R system is predominantly part of immune effector cell functioning and has variable effects on tumor progression and may differentially impact distinct stages of tumor development.

### Immune Effector Cells

Immune effector cells from both the innate and adaptive immune system are part of the TME and elicit both pro- and antitumorigenic responses. Various immune effector cells, described in more detail below, express TRAIL allowing them to bind and activate TRAIL-Rs on tumor cells.

NK cells are the main effector cells from the innate immune response and eliminate aberrant tumor cells by granule release (perforin/granzyme) dependent toxicity and via membrane receptor interactions involving FasL, TNFα, and TRAIL depending on their differentiation and activation status (30, 31). In syngeneic cancer mice models activation of NK cells by IL12 resulted in IFNγ production, which was essential for further activation and augmenting TRAIL surface expression responsible for anti-metastatic activity in TRAIL sensitive tumors (31, 32). Depletion of each single component of the NK cell-IFNγ-TRAIL axis promoted tumor growth in a chemical-induced murine sarcoma model illustrating its importance in antitumor responses (33). Moreover, activated NK cells employ membrane bound TRAIL, but not soluble TRAIL, to support their cytotoxicity against neuroblastoma cells, which are normally resistant to soluble TRAIL (34).

Cytotoxic T-cells (CTLs) are the main effector cells of the acquired immune response and also make use of the TRAIL/TRAIL-R system to induce apoptosis in target cells. For example, expression of TRAIL on CTLs can be enhanced by T-cell receptor-mediated interaction with TRAIL-R-expressing human non-small cell lung cancer (NSCLC) cells. IFNα significantly enhanced TRAIL expression on CTLs and effectively triggered apoptosis in TRAIL sensitive NSCLC cells *in vitro*. Antitumor activity was also seen in immune–deficient mice implanted with TRAIL sensitive NSCLC cells in which intratumoral injection of autologous activated CTLs resulted in TRAIL-R2-dependent tumor cell death (35).

DCs play a role in both innate and adaptive immune responses by communicating to both immune effector cells and presenting antigens to T cells. Cytotoxic DCs can be activated by IFNα or IFNγ displaying antitumor activity adopting the TRAIL/TRAIL-R system (36, 37).

The activity of the immune effector cells can be counteracted by immunosuppressive Tregs. Tregs secrete a range of soluble factors such as TGFβ, IL10, and IL35, which can suppress effector T cell expansion and cytokine secretion (IFNγ, TNFα) (38). Tumor infiltrating Tregs repress antitumor immune responses by inhibiting the cytotoxic activity of CTLs, NK cells, and DCs. In rodent colon cancer models Tregs were able to inhibit cell death induced by TRAIL expressing DCs. Innate immune response activation by Mycobacterium Bacillus Calmette-Guérin (BCG) combined with cyclophosphamide treatment depleted Tregs and potentiated DC-induced tumor cell killing (39). Orthotopic implantation of TRAIL resistant murine pancreatic cancer cells in either WT or TRAIL knockout mice resulted in smaller tumors in TRAIL knockout mice. Tumor growth in WT mice was associated with increased tumor infiltrating CD4^+^ Treg cells that was further enhanced by treating mice with recombinant TRAIL, which also enhanced tumor growth. Although the underlying mechanism of TRAIL-dependent tumor infiltration was not addressed, it is likely that TRAIL in the context of resistant tumor cells potentiated the immune suppressive effects of Treg cells resulting in enhanced tumor growth (40). Notably, in mice Tregs can also directly eliminate CTLs via TRAIL/TRAIL-R2-mediated apoptosis (41). On the other hand, CTLs can produce cytokines that increase the sensitivity of tumor cells for TRAIL. Upon T-cell receptor activation CD8+ CTLs produced soluble IFNγ and TNFα, which increased the susceptibility of neuroblastoma cells for TRAIL-induced caspase-8 activation (42).

Tumor cells can counteract the activity of immune effector cells by inhibiting TRAIL-induced apoptotic signaling. For example, follicular lymphomas expressing CD40, an important co-stimulatory receptor able to interact with ligand expressed on germinal center CD4^+^ T-cell subpopulations, protected from TRAIL-induced apoptosis by CTLs. CD40 activation induced NF-κB leading to upregulation of antiapoptotic cFLIP and Bcl-XL (43, 44).

Antitumor activity of TRAIL expressing DCs was reduced by apoptotic tumor lysate derived from TRAIL sensitive murine lymphomas. TRAIL expression could be partially restored both *in vitro* and *in vivo* upon stimulation by IL15, or LPS leading to prolonged TRAIL expression on DCs and antitumor activity. On the other hand, while stimulating DCs, IL15 inactivated STAT3 in lymphoma cells resulting in TRAIL resistance that could be neutralized by combined treatment with the STAT3 inhibitor Cucurbitacin I leading to an overall effective therapeutic response (45).

Activated TRAIL-Rs on tumor cells can also trigger a counterattack and create an immune suppressive TME. TRAIL resistant human colon cancer cells were found to release microvesicles containing FAS and TRAIL that were also detectable in plasma from patients. These microvesicles could eliminate CTLs thus providing an immune escape mechanism (46). Endogenous cell surface TRAIL on multiple myeloma cells could eliminate osteoclasts and prevent bone formation thereby facilitating metastatic lesions (47, 48). TRAIL-resistant gastric carcinoma cells from primary and metastatic patients expressed TRAIL and TRAIL-Rs, including TRAIL-R4. Interestingly, tumor infiltrating lymphocytes (TIL) from patients with a primary tumor hardly expressed TRAIL/TRAIL-Rs, whereas those from metastatic patients showed high levels and displayed apoptosis. This suggests that in metastatic lesions tumor cells can evade immune surveillance by inducing TRAIL- mediated cell death of TILs (49). Similarly TRAIL expressing colorectal cancer (CRC) cells in patient samples were linked with apoptosis induction in tumor infiltrating CD8^+^ T-cells via TRAIL-R1 providing an immune escape mechanism (50).

Taken together, the TRAIL-dependent immune effector function can be potentiated by various cytokines that can be counteracted by Tregs. Particularly TRAIL resistant tumors, but also sensitive tumor cells can respond by expressing or secreting factors that inhibit immune effector cell-induced apoptosis or even eradicate immune cells by TRAIL/TRAIL-R dependent mechanisms.

### Neutrophils and Macrophages

Neutrophils are an essential part of the innate immune system and are the most abundant leukocytes in the blood. Similar to monocytes that can differentiate into macrophages, neutrophils possess phagocytic activity. They can migrate to sides of acute inflammation as well as tumors where they can have both tumor suppressive and supportive functions (51). Neutrophils and monocytes both express TRAIL and target TRAIL-R expressing tumor cells. *In vitro* experiments showed that IFNα exposure of neutrophils/monocytes led to increased release of soluble TRAIL resulting in apoptosis activation in TRAIL sensitive chronic myeloid leukemia (CML) cells. Additionally, IFNα protected both neutrophils and monocytes from leucine-zipper TRAIL and soluble rhTRAIL induced apoptosis, which may be related to absence or low levels of TRAIL-R1/R2 and high TRAIL-R3 expression. Furthermore, melanoma patients treated with IFNα showed increased soluble TRAIL serum levels indicating *in vivo* relevance of this antitumorigenic mechanism (52). This mechanism provides an explanation for treatment efficacy of IFNα in CML and melanoma patients.

Peritumoral administration of granulocyte colony-stimulating factor (G-CSF) has been reported to suppress murine mammary adenocarcinoma progression in mice, which was not seen upon *in vitro* exposure of tumor cells to this cytokine. G-CSF appeared to increase the number of infiltrating neutrophils accompanied with upregulation of death-inducing proteins including TRAIL providing an explanation for antitumor activity (53).

In esophageal squamous cell carcinoma (ESCC) the presence of IL17 producing cells was associated with a favorable prognosis. IL17 stimulated ESCC-dependent secretion of neutrophil-attracting chemokines and, moreover, enhanced their immune effector function also characterized by TRAIL (54).

Another study found that cathepsin E expressed on immune cells can cleave and activate endogenous cell surface TRAIL on prostate and melanoma cells and enhance macrophage infiltration leading to antitumor activity. Cathepsin E reduced murine melanoma growth in mice, when compared to tumor growth in cathepsin E knockout mice. This was accompanied with increased tumor infiltration of activated macrophages and apoptosis activation in tumor cells (55).

Macrophages were reported to secrete matrix metallopeptidase 12 (MMP12) and stimulate TRAIL-dependent apoptosis in tumor cells. MMP12 activity could be mimicked by a recombinant C-terminal domain peptide, named SR20, that could induce TRAIL-mediated apoptosis both in oncogenic mutated KRAS and WT murine and human NSCLC cells as demonstrated *in vitro* and in orthotopically implanted mice and a KRAS-induced murine mouse model. In addition to protein cleavage activity, SR20 translocated to the nucleus of these cells leading to transcriptional upregulation of *TRAIL* and *TRAIL-R1* mRNA and downregulation of antiapoptotic proteins that was responsible for the observed tumor cell death (56).

Conversely, in addition to the TRAIL-dependent antitumor activity of neutrophils and macrophages also protumorigenic activity has been demonstrated involving the cooperative action of various cytokines produced by tumor cells and different immune cells. For example, in murine hepatocarcinoma and melanoma mice models IL35 was found to polarize neutrophils into a protumorigenic N2 state and enhance tumor infiltration that was accompanied by downregulation of TRAIL expression. This involved the concerted action of various cytokines and immune cells cumulating in IL6/IL1β/IL17/G-CSF induced STAT3-dependent downregulation of TRAIL expression on neutrophils and simultaneous upregulation of MMP9 together resulting in immune suppression and a proangiogenic state (57, 58).

The MUC5AC glycoprotein expressed on pancreatic cancer cells was required for tumor growth *in vivo* by suppressing antitumor effects of neutrophils. MUC5AC was found to suppress tumor secretion of the neutrophil attractant IL8 and, moreover, MUC5AC blocked TRAIL-R mediated apoptosis of tumor cells via an as yet unknown mechanism (59).

Summarizing, macrophages and neutrophils can eliminate tumor cells via the TRAIL/TRAIL-R system that can be potentiated or suppressed by various mechanisms involving administrated or tumor-derived cytokines as well as tumor or immune cell expressed activators or suppressors.

### Non-hematopoietic Stromal Cells

Expression of TRAIL on stromal cells has been demonstrated to be a favorable characteristic for patient survival (60). Immunohistochemistry (IHC) studies of patient tissue arrays demonstrated that increased TRAIL expression in the epithelium and connective tissues of prostate and ovarian cancer is associated with elongated recurrence free survival and favorable overall survival, respectively. This effect was independent of decreased TRAIL-R expression and increased cFLIP-L expression in tumor cells (61, 62).

Mesenchymal stem cells (MSC) are derived from bone marrow and can differentiate into various cell types including osteoblasts and adipocytes and home to tumors making part of the tumor stroma. *Ex vivo* exposure of human MSCs to TNFα increased TRAIL expression. Subsequent infusion of these hMSC in mice implanted with MDA-MB-231 breast cancer cells inhibited tumor growth. Furthermore, co-culturing these hMSC with several cancer cell lines resulted in apoptosis induction. Interestingly, these dying tumor cells released DNA that acted as damage-associated molecular patterns (DAMPs) that via a TLR3-dependent NF-κβ feed forward loop further increased TRAIL expression on hMSC, thereby potentiating their antitumor activity (63). In follow up work TNFα-activated hMSC were also found to produce IFNβ in response to released DNA/RNA from apoptotic breast cancer cells further enhancing TRAIL expression and potentiation of antitumor activity (64). Accordingly, this feedforward loop of TRAIL-induced apoptosis was not seen in apoptosis resistant breast cancer cells. Moreover, CAFs isolated from breast cancer patients showed a similar increase in TRAIL and IFNβ upon exposure to DNA.

Thus, bidirectional signaling between TRAIL sensitive tumor cells and stromal cells can create a tumor-suppressive TME.

## Modulation of Exogenous TRAIL Receptor Agonist Activity and The TME

From a therapeutic standpoint pharmacological administration of TRAIL-R agonists aims to mimic the function of TRAIL-expressing immune effector cells. In this part the impact of different components of the TME on the efficacy of exogenously administrated TRAIL is highlighted.

### Stromal Cells

Stromal cells express TRAIL decoy receptors and create a sink for administrated recombinant TRAIL leading to suppression of antitumor activity. OPG is predominantly secreted by osteoblasts and functions as a paracrine survival factor in bone marrow TME and has been implicated in TRAIL resistance. OPG protected prostate cancer and multiple myeloma cells against TRAIL-mediated cell death (65, 66). Bone marrow stromal cells from breast cancer patients also produced sufficient OPG levels to decrease TRAIL sensitivity of breast cancer cells providing a mechanism for the occurrence of metastatic lesions in the bone (67). OPG production can be enhanced by cytokines. For example, IL1β increased OPG expression in both TRAIL sensitive MDA-MB231 and resistant MCF7 cells. Gene silencing of OPG enhanced apoptosis in MDA-MB231 cells, but not in MCF7 cells, and a positive correlation was found between OPG levels and TRAIL sensitivity (68). TRAIL variants have been developed with reduced affinity for decoy receptors and demonstrated superior antitumor activity in the presence of OPG producing cells or recombinant OPG (69). Similarly, the expression of TRAIL-R3/R4 on CAFs decreased the efficacy of TRAIL induced apoptosis in tumor cells that could be bypassed by developed TRAIL variants with reduced binding activity to these decoy receptors (70).

Epidermal growth factor (EGF) could protect TRAIL sensitive HEK293 and MDA-MB-231 tumor cells for apoptosis involving Akt activation and inhibition of mitochondrial apoptosis (71). On the other hand, in non-transformed MCF10A human breast epithelial cells EGF sensitized for TRAIL-induced apoptosis that was counteracted by TGFβ involving reduction of DISC formation and activation of cytoprotective autophagy (72). The differential activity of EGF in normal and tumor cells illustrates differential wiring of TRAIL signaling upon oncogenic transformation.

Cytokines exogenously added or produced by either tumor or stromal cells can inhibit TRAIL-induced apoptosis in tumor cells. TRAIL-sensitive ovarian cancer cells reverted to resistant cells by exposure to IL8 that was associated with downregulation of TRAIL-Rs (73). IL8 produced by tumor cells or recombinant IL8 also was shown to suppress TRAIL-induced apoptosis in prostate cancer cells by up-regulation of the anti-apoptotic proteins cFLIP(S) and cFLIP(L) in a CXCR2 and NF-κB-dependent way. TRAIL as well as chemotherapy could enhance IL8 expression leading to apoptosis resistance and a CXCR2 antagonist sensitized for TRAIL providing a therapeutic strategy (74). Primary cancer cells from breast, colon, and lung carcinomas produce IL4 that protected tumor cells for TRAIL-induced apoptosis by increasing expression of a number of anti-apoptotic proteins including cFLIP, Bcl-XL, and Bcl-2 (75). Metastatic melanoma cells endogenously express proinflammatory TNFα and IL6 leading to constitutive NF-κB, STAT3, and COX2 expression. Neutralizing antibodies against these cytokines and genetic or pharmacological inhibition of the downstream pathways resulted in sensitization for exogenous TRAIL-induced apoptosis (76). TLR4 ligation on human lung cancer cells and associated NF-κB activation reduced apoptotic effects of TRAIL and, in addition, promoted the production of immunosuppressive cytokines TGFβ and IL8 together with proangiogenic VEGF (77).

Ovarian cancer (OC) is commonly associated with peritoneal ascites production and provides a unique TME for this tumor type. A proportion of ascites samples taken from OC patients could mediate resistance toward TRAIL-induced apoptosis in a panel of OC cell lines *in vitro* (78). Further research showed that malignant ascites leads to activation of PI3K, Akt, ERK1/2, and ELK1 and up-regulating cFLIP(s) and Mcl-1 and inhibition of TRAIL-induced caspase-dependent apoptosis (79).

Co-culturing of multiple myeloma cells and HS5 stromal cells attenuated TRAIL-induced cell death involving soluble factors produced by the stromal cells. Antiapoptotic cFLIP was identified as a mediator for resistance as silencing its expression increased TRAIL sensitivity (80). In a follow up study stromal-mediated resistance was found to involve NF-κB-dependent cFLIP expression that could be prevented by the proteasome inhibitor bortezomib that restored TRAIL sensitivity in tumor cells without affecting stromal HS5 cells (81). Myoblasts secreting platelet-derived growth factor BB (PDGF-BB) indirectly affected TRAIL sensitivity by activating Hedgehog (Hh) signaling in cholangiocarcinomas (CCA) thereby shifting the cells toward TRAIL resistance. Inhibition of Hh signaling by cyclopamine increased apoptosis in CCA cells *in vitro* and in a syngeneic RAT CCA model resulting in tumor suppression (82). Co-culturing of Wnt producing rat embryonic fibroblasts protected TRAIL sensitive human pre-B leukemia cells for TRAIL-induced apoptosis. Although the precise mechanism was not fully elucidated inhibition of MEK1/ERK1/2 and NF-κB signaling sensitized for TRAIL (83).

TRAIL resistant colon cancer cell lines were sensitized for exogenously administrated TRAIL by combined exposure with IFNγ and TNFα through down-regulation of Bcl-XL. Evidence for a similar resistance mechanism was provided in a murine CT26 colon carcinoma mice model. In this model, tumor infiltrating macrophages, NK cells and T cells secreting IFNγ and TNFα and expressing TRAIL were responsible for suppression of lung metastases since neutralizing TRAIL antibodies blocked antitumor activity leading to increased lung metastases. Moreover, it was shown that adoptive transfer of tumor-specific CD8+ CTLs producing IFNγ and TNFα together with recombinant TRAIL/agonistic mAb therapy effectively induced apoptosis in CT26 tumor cells in mice, whereas TRAIL alone was ineffective, indicating cooperative activity between tumor infiltrating immune cells and TRAIL therapy (84).

Taken together, stromal cells can produce various factors that in a paracrine way suppress or enhance the therapeutic efficacy of TRAIL-R agonists.

### TME Remodeling

Application of exogenous TRAIL may also impact the TME by targeting specific cellular components. In contrast to the dogma that normal cells are refractory to the death inducing effect of TRAIL Liguori et al. reported susceptibility of monocytes and macrophages for TRAIL-induced apoptosis (85). *In vitro*, human monocytes and macrophages expressed TRAIL-R1/R2 and underwent apoptosis after TRAIL exposure, whereas neutrophils and lymphocytes expressing mainly decoy TRAIL-R3 did not. Furthermore, in murine fibrosarcoma implanted mice, TRAIL-R expressing monocytes were sensitive for TRAIL. Interestingly, particularly infiltrating TAMs but not normal tissue resident macrophages expressed functional TRAIL receptors. TAMs that have protumorigenic activity in this syngeneic mouse model were sensitive to TRAIL-induced apoptosis resulting in significant decreases in circulating monocytes and infiltrating TAMs and a concomitant reduction in tumor growth and metastasis (85).

TRAIL resistant murine hepatocellular carcinoma (HEPA-1-6) cells *in vitro*, became sensitive for intratumoral injection of TRAIL after implantation in mice. Analyses of tumor infiltrating immune cells revealed that TRAIL injections decreased the numbers of Trail-R positive Tregs, whereas levels of CD8^+^ CTLs increased. Thus, TRAIL treatment appears to deplete Tregs by apoptosis thereby potentiating CD8^+^ CTLs-dependent antitumor responses, in addition to the direct apoptotic effects on HEPA-1-6 tumors (86).

In acute myeloid leukemia HL-60 cells TRAIL triggered apoptosis. However, in the surviving fraction an increase in monocyte maturation markers was observed, requiring TRAIL-R1 and caspases activation. In normal monocytes TRAIL also was able to induce expression of CD14 and CD11b maturation markers associated with enhanced phagocytic capacity and antitumor activity. Accordingly, TRAIL therapy has dual anti-neoplastic activity by directly killing tumor cells and enhancing monocyte/macrophage activity (87).

TRAIL could stimulate the production of pro-inflammatory cytokines IL1β, IL6, and TNFα in a NF-kB-dependent way in human and murine macrophages *in vitro*. Similarly, TRAIL was able to stimulate pro-inflammatory cytokine expression in TAMs in tumors derived from TRAIL sensitive H460 NSCLC cells in nude mice, but not in peritoneal macrophages that was related to high miR-146 expression in the latter leading to silencing of cytokine expression. Moreover, co-cultures of H460 and TRAIL-stimulated TAMs showed that cytokines produced by TAMs potentiate the TRAIL-dependent killing of H460 cells (88).

Tumor vasculature has been reported to be sensitive for TRAIL-induced apoptosis via TRAIL-R2. In different murine tumor models tumor-associated endothelial cells expressed TRAIL-R2 and were sensitive for the killing effect of TRAIL resulting in tumor starvation, even when tumor cells were TRAIL resistant. IHC demonstrated TRAIL-R2 expression in NSCLC patient vasculature and therefore TRAIL-induced collapse of tumor blood vessels was proposed as an alternative or complementary therapeutic strategy (89). Another favorable effect of TRAIL on the TME was reported by downregulating OPG production in MSCs, fibroblasts and endothelial cells by TRAIL-mediated inhibition of p38/MAPK activation (90).

These findings illustrate that the antitumor activity of TRAIL can be directly enhanced by simultaneously potentiating the antitumor effect of stromal cells or by suppressing the protumorigenic activity of stromal cells leading to an overall therapeutic benefit.

Contrary to above findings, tumor cells were found to create a protumorigenic niche involving TRAIL signaling via indirect means. In TRAIL resistant NSCLC cells TRAIL exposure triggered the secretion of immune-suppressive cytokines IL8, CXCL1, CXCL5, and CCL2 in a FADD- and caspase-8-dependent way. Particularly CCL2 was found to induce monocyte polarization into MDSCs and generated M2 macrophages. Orthotopic implanted TRAIL resistant A549 NSCLC and murine LL3 cells with FADD deleted showed reduced tumor growth compared to implanted WT tumor cells. This was associated with decreased cytokine production including CCL2 leading to reduced tumor infiltration of tumor supporting monocytes and MDSCs (91).

TRAIL treatment of resistant human pancreatic cells resulted in enhanced invasion *in vitro* and metastatic spread in orthotopically implanted nude mice. TRAIL-induced NF-κB activation stimulated production of pro-inflammatory cytokines IL8 and MCP1/CCL2, proteases MMP7 and MMP9 and urokinase-type plasminogen activator (uPA) that were responsible for pro-inflammatory effects and metastatic spread (92, 93). More recently, the chemokine CCL20 was identified as a TRAIL/NF-κB inducible target gene in resistant pancreatic cancer cells that indirectly modulated TRAIL resistance in mice by recruiting peripheral blood mononuclear cells (PBMCs), which further increased TRAIL resistance of CCL20-producing pancreatic cancer cells (94). Thus, in pancreatic cancer TRAIL had unfavorable effects by stimulating pro-inflammatory cytokines production leading to enhanced metastasis and TRAIL resistance.

A positive feedback loop between tumor cells and macrophages was identified in promoting growth and survival of colon cancer cells. Macrophages producing IL1β could stimulate growth of colon cancer cells by activating GSK3β/Wnt signaling (95). IL1β production by macrophages was induced by tumor cells and resulted in protection of colon cancer HCT116 cells from TRAIL-induced apoptosis. IL1β-mediated TRAIL resistance involved activation of NF-κB and GSK3β/Wnt pathways leading to stabilization of the EMT transcription factor Snail (96).

TRAIL treatment was also shown to enhance pro-inflammatory cytokine and chemokine production, including IL6, IL8, MCP1, CXCL1, and MIF, in various cancer cell types independent from TRAIL sensitivity although cytokines levels were higher in resistant cancer cells. MCP1 promoted chemotaxis of THP-1 monocytes and IL8 recruited neutrophils that may enhance tumor growth. Mechanistic studies revealed that caspase-8 was required for both apoptosis activation and cytokine production, although non-cleaved procaspase-8 was responsible for cytokine production by functioning as a scaffold for formation of a FADD-Caspase-8-RIPK1-TAK1 signaling complex and subsequent activation of the MEK/ERK pathway and cytokine production (97).

Taken together, depending on the experimental model and tumor type the overall antitumor effect of exogenous TRAIL is modulated by paracrine effects elicited either by direct activation of TRAIL-Rs on tumor cells or indirectly by activation of TRAIL-Rs on stromal cells, particularly immune cells. These effects can have either positive or negative impact on antitumor activity or even have tumor promoting effects.

## Biochemical and Biophysical Properties of the TME

Besides cellular compounds, the TME is also characterized by hypoxia, increased acidity and aberrant tissue stiffness involving alterations in ECM as well as aberrant interstitial pressure. Consequences for TRAIL signaling are exemplified below.

### The ECM

The ECM is a collection of different macromolecules that are assembled in a three-dimensional structure with unique biochemical and biomechanical properties regulating cell growth, survival, motility, and differentiation. The ECM provides cells with a scaffold and regulates hydration and pH as well as the availability of growth factors and cytokines (98). Cell-ECM interactions play an important role in tumor development and maintenance, and degradation of the ECM is associated with metastatic spread of tumor cells. ECM-cell adhesion signaling predominantly involves interactions between cell surface integrins and fibronectin. Genetic and pharmacological targeting of Integrin-β and downstream signals such as Src, Talin, PI3K, and MAPK sensitized both apoptosis resistant and sensitive tumor cells for TRAIL-induced apoptosis *in vitro* by increasing TRAIL-R leveling and reducing the threshold for mitochondrial apoptosis (99). These findings also provide a mechanistic rationale for adherent tumor cells being more resistant to TRAIL than disseminating cells. On the other hand, loss of the epithelial adhesion protein E-cadherin, a key characteristic of cells undergoing EMT, has been linked with TRAIL resistance. EMT induction in lung cancer cells resulted in TRAIL resistance and silencing of E-cadherin also inhibited apoptosis activation. Mechanistically, E-cadherin was found to bind to ligated TRAIL-R1and/or TRAIL-R2 and augment their clustering and coupling to the actin cytoskeleton resulting in efficient DISC assembly and caspase-8 activation. Although elevated levels of E-cadherin in a panel of tumor cells correlated with TRAIL sensitivity, ectopic overexpression of E-cadherin in TRAIL resistant tumor cells did not lead to sensitization indicating context dependency (100).

Other components of the ECM were also found to regulate TRAIL sensitivity. The elastin microfibril interface-located protein 2 (EMILIN2), a member of the family of ECM glycoproteins, can bind to TRAIL-R1 and to a lesser extent TRAIL-R2 to induce receptor clustering and co-localization in lipid rafts subsequently activating apoptosis (101). The CCN family of integrin-binding matricellular proteins have pleiotropic functions including regulation of cell proliferation and survival. In prostate cancer cells CCN1 was reported to support cell adhesion via integrins and heparan sulfate proteoglycans (HSPG) and promote growth. However, CCN1 also led to sensitization to TRAIL-induced apoptosis that was dependent of CCN1 interations with integrins and HSPG receptor Syndecan-4 and activation of protein kinase C (102). Thus, the ECM can modulate TRAIL-R functioning and affect the outcome of TRAIL exposure.

### Mechanical Stress

Tumor cells experience elevated mechanical stress as a result from multiple factors including increased interstitial fluid pressure by aberrant vasculatures and lack of functional lymphatic vessels in tumors. Moreover, these cells experience solid stress as a result of tumor cell proliferation, aberrant ECM production, and an altered TME. Together these factors result in enhanced mechanical stress in tumors that has been associated with decreased efficacy of anti-cancer treatment (103).

A limited number of studies examined the role of mechanical stress on TRAIL sensitivity. Elevated pressure on hepatoma Hep3B cells was reported to sensitize for TRAIL-induced apoptosis by suppressing ERK1/2 activation resulting in decreased Bad phosphorylation and enhanced mitochondrial apoptosis (104). Similarly, elevated atmospheric pressure on H460 NSCLC cells enhanced TRAIL-dependent apoptosis associated with up-regulation of TRAIL-R2 and potentiation of caspase-8, accompanied by enhanced c-FLIP degradation and reduced expression of XIAP and the antiapoptotic protein Survivin. Enhancement of TRAIL-induced apoptosis was also detected in additional cancer cell lines, whereas normal fibroblast remained TRAIL resistant (105). The mechanism transmitting pressure-induced TRAIL sensitization has not been elucidated yet.

Interestingly, in xenograft mice models TRAIL treatment rapidly reduced interstitial fluid pressure in TRAIL sensitive tumors, but not in TRAIL resistant ones that was accompanied by less condensed tumors. TRAIL treatment was associated with changes in the TME including stromal widening, macrophage infiltration, and better vascular perfusion, which also increased the efficacy of chemotherapy efficacy (106). Another study in larynx carcinoma HEP2 cells showed a correlation between stiffness and inhibition of TRAIL-induced apoptosis. Actinomycin D treatment reduced cellular stiffness that was linked with F-actin depolarization and susceptibility for TRAIL dependent apoptosis involving decreased Bcl-2 expression (107).

Indirect evidence for a suppressive effect of mechanical stress on TRAIL-mediated apoptosis was provided by Cho et al. (108). Lung tumor stroma is enriched for fibronectin, which is a multimodular protein able to stretch by partially unfolding under mechanical pressure. This stress can be experimentally mimicked by administration of recombinant type III domain of fibronectin (FnIII-1c). FnIII-1c reduced TRAIL sensitivity in H460 lung cancer cells. Inhibition of TRAIL induced caspase-8-dependent apoptosis was mediated by PI3K/Akt pathway activation via increased binding of αvβ5 integrin to its ligand vitronectin, a plasma protein and ECM factor (108).

Together, few studies thus far showed mostly a positive effect of mechanical stress on TRAIL-induced apoptosis, although clearly more research is required.

### Hypoxia

Hypoxia is a common condition of the TME and has been reported to affect TRAIL signaling by a variety of mechanisms leading to either enhanced or reduced TRAIL sensitivity. Most studies found that a hypoxic TME diminishes TRAIL sensitivity by a diversity of mechanisms. For example, hypoxia could block mitochondrial apoptosis by upregulating anti-apoptotic proteins or downregulating pro-apoptotic members of the BCL-2 family (109). More recently, hypoxia was shown to stimulate mitochondrial autophagy resulting in impairment of the mitochondrial amplification loop by reducing mitochondrial release of pro-apoptotic factors such as SMAC. Exogenous substitution by SMAC mimetics or inhibition of XIAP restored TRAIL induced apoptosis under hypoxic conditions (110). The hypoxia-inducible transcription factor HIF1α was found to be essential for hypoxia-dependent inhibition of TRAIL-induced apoptosis in a number of cancer cell lines *in vitro* (111). Expression of the cellular prion protein (PrPc) was shown to be enhanced by HIF1α under hypoxia and to mediate TRAIL resistance in colon cancer cells *in vitro* and *in vivo* and may involve enhanced Akt and Bcl-2 activity (112). Another study linked HIF1α as a major mediator of enhanced TRAIL-R4 production, but not other TRAIL-Rs, at the cell surface of colon cancer cells (113). HIF2α that has been less well-studied in context of TRAIL signaling had a protective effect on TRAIL-induced apoptosis in most pancreatic cancer cell lines tested by transcriptionally enhancing the expression of antiapoptotic protein Survivin. Accordingly, the Survivin inhibitor YM155 sensitized for TRAIL apoptosis under hypoxia (114).

Although most studies demonstrated a decrease in TRAIL-driven cell death under hypoxic conditions, several studies reported pro-apoptotic activity. For example, hypoxia increased TRAIL-induced apoptosis in DU-145 and LNCaP prostate cancer cells accompanied by enhanced activation of caspase-8 and−3 but not caspase-9 (115). In breast cancer cells hypoxia increased TRAIL-R2 expression via JNK and C-Jun resulting in increased TRAIL sensitivity (116). Others found that hypoxia decreased PKCε levels in a HIF1α dependent way leading to sensitization for TRAIL (117). The underlying cause of the differential effects of hypoxia on TRAIL sensitivity is unclear, but may be related to the degree in which the cancer cell line tested is dependent on activation of the mitochondrial amplification loop (type-II cells).

### Extracellular pH

High dependency of tumor cells on glucose to fuel aerobic glycolysis, known as the Warburg effect, provides energy and biosynthetic metabolites required for growth. Hypoxic conditions favoring anaerobic glycolysis in tumor cells further enhances the production of extracellular lactate and is a main cause of an acidic pH in the TME (118). TRAIL was found to induce cell death in a pH-dependent manner. At low pH (6.6) TRAIL-induced apoptosis was augmented in prostate carcinoma and colorectal carcinoma characterized by increased tBID/BAX interactions, cytochrome C release and caspase activation (119). In gastric carcinoma cells low pH resulted in upregulation of TRAIL-R1 and –R2 gene and protein expression and increased proapoptotic activity of TRAIL via TRAIL-R2 (120). Others reported that an acidic extracellular pH of 6.5 enhanced TRAIL-induced cell death in colon carcinoma and hepatocarcinoma cell lines by switching to activation of a caspases- and RIPK1-dependent necroptosis (121).

### Glucose

Tumor cells suffer usually from glucose deprivation and hypoglycemic conditions, which can impact TRAIL signaling. TRAIL sensitivity was increased in a glucose deprived environment in a variety of cancer cell lines (122, 123). Glucose deprivation enhanced TRAIL sensitivity by increasing DISC formation and potentiation of mitochondrial depolarization and cytochrome c release and subsequent caspase activation (115, 122, 123). In addition, glucose deprivation also increased ceramide levels leading to inhibition of Akt and reduced cFLIP levels leading to enhanced TRAIL sensitivity (123).

In mantle cell lymphoma cells chronic glucose deprivation resulted in a switch from aerobic glycolysis to oxidative phosphorylation thereby maintaining ATP production that was accompanied by reduced sensitivity toward TRAIL induced apoptosis. Glucose-free conditions led to decreased surface expression levels of TRAIL-R1/R2, impaired DISC formation, increased levels of Bcl-2 and XIAP, decreased levels of Bax and cytosolic cytochrome c. Conversely, 2-deoxyglucose that inhibits glycolysis and caused a reduction in ATP levels sensitized for TRAIL-induced apoptosis by potentiating DISC-dependent caspase-8 apoptosis as a result of a general decrease in mRNA translation including antiapoptotic proteins such as cFLIP. This study indicated differences between chronic and temporally glycolysis inhibition on TRAIL signaling likely related to mitochondrial functioning and intrinsic apoptosis (124). In a follow up study by the same group the balance between the Akt and AMPK and downstream regulation of mTORC1 was proposed to be instrumental in modulating protein translation and the equilibrium between pro- and anti-apoptotic Bcl2 family members (125).

Taken together, mechanical stress, hypoxia, pH, and glucose availability all have a direct effect on tumor cell apoptosis sensitivity for TRAIL. The impact of these conditions on stromal cells, and their indirect effects on tumor cells have not been examined as yet.

## Conclusions and Future Directions

To unleash the full clinical potential of TRAIL receptor agonists we need to unravel the complexity of TRAIL signaling pathways in order to effectively bypass apoptosis resistance. As illustrated here, the TME plays an important role in modulating the efficacy of both the endogenous TRAIL/TRAIL-R system mostly used by immune cells as well as of exogenously administrated therapeutic TRAIL receptor agonists. This modulation is complex involving a multi-component TME and a variety of often bidirectional signals that regulate TRAIL-driven apoptosis at distinct cellular and molecular levels. In this context the tumor model and experimental conditions used are of key importance, giving rise to different outcomes of TRAIL/TRAIL-R signaling, being either tumor promoting or suppressive effects. The use of syngeneic mouse models with a fully active immune system appear most valuable to dissect TME-cancer interactions, although obviously the TRAIL/TRAIL-R system in humans is not identical to that in mice.

**Figure 1:**
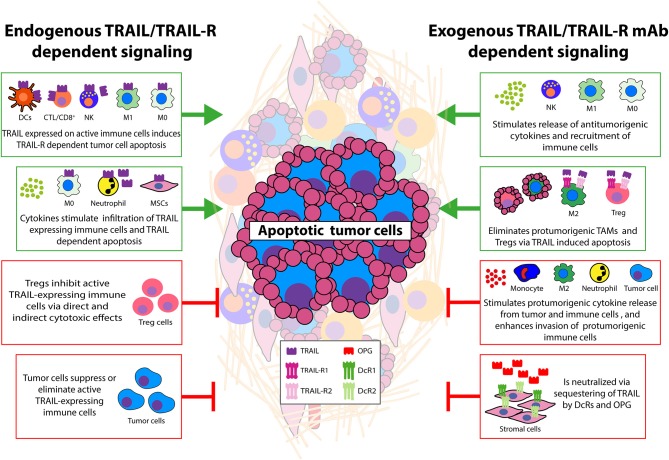
Main interactions between tumor and cellular TME that modulate TRAIL signaling. Schematic overview of effects of cellular TME—tumor interactions on TRAIL/TRAIL-R signaling. Green boxes depict antitumorigenic TME interactions. This can be achieved by endogenous TRAIL expression on activated immune effector cells leading to TRAIL-R dependent apoptosis in tumor cells. TRAIL-expressing neutrophils and macrophages can also eliminate tumor cells via TRAIL-Rs and cytokines can enhance infiltration of these cells potentiating tumor killing. Other stromal cell types expressing TRAIL may also display antitumor activity. Exogenous recombinant TRAIL, or TRAIL-R agonistic antibodies can induce cell death in tumor cells and immune suppressive cells (TAMs, Tregs) resulting in enhanced numbers of CTLs and increased phagocytic capacity of neutrophils/monocytes/macrophages. TRAIL-induced cell death of tumor endothelial cells has also been demonstrated (not depicted). Exogenous TRAIL can stimulate release of cytokines able to further increase TRAIL/TRAIL-R levels on immune effector cells. Together these events potentiate antitumor activity via the TRAIL/TRAIL-R system. Red boxes depict protumorigenic interactions. Endogenous TRAIL/TRAIL-R expression on often resistant tumor cells can induce TRAIL-driven cell death in antitumor TILs, CTLs. Cytokines can down-regulate expression of TRAIL on immune effector and neutrophils/macrophages. Decoy TRAIL-R3/R4 and OPG expression on stromal and tumor cells can sequester exogenously added TRAIL/TRAIL-R agonists and protect against apoptosis. Resistant tumor cells stimulated with TRAIL secrete cytokines that recruit immune suppressive cells and/or induce a suppressive phenotype in tumor infiltrated cells. Cytokines derived from tumor and stromal cells can increase tumor TRAIL resistance by enhancing antiapoptotic signaling or even stimulate metastasis. Although often studied separately, it is anticipated that TME-dependent modulation of endogenous and exogenous TRAIL activity will occur simultaneously. See text for details.

The cellular TME, consisting of among others immune effector cells, immune-suppressive Tregs, neutrophils, macrophages, and non-hematopoietic stromal cells, is able to enhance or reduce the antitumor activity of TRAIL-expressing immune effector cells as well as of exogenous TRAIL (see also Figure 1). In response to TRAIL-R activation tumor and stromal cells can initiate feed forward mechanisms or launch a counterattack leading to suppression of antitumor activity. Interestingly, therapeutic TRAIL can also remodel the TME by, for example, eliminating tumor-infiltrating macrophages, Tregs, or tumor endothelial cells resulting in additional antitumor activity. Conversely, TRAIL-R stimulation of particularly apoptosis resistant cancer cells can have protumorigenic effects, illustrated by enhanced cytokine secretion and attraction of immune suppressive cells. Although the underlying mechanisms need further clarification, regulation of endogenous and exogenous TRAIL sensitivity by tumor—stroma cell interactions frequently involve regulation of TRAIL/TRAIL-R levels including decoy receptors and OPG, and NF-κB-dependent regulation of intracellular pro- and antiapoptotic factors such as Bcl2 family members and IAPs together with secretion of cytokines in feedforward or feedback loops. In some studies the efficacy of therapeutic TRAIL could be potentiated by inhibiting these antiapoptotic regulators. In addition, sequestering of TRAIL by upregulation of decoy receptors can be minimized by the use of designed recombinant TRAIL variants or TRAIL-R1 or -R2 agonistic antibodies that have strongly reduced binding affinity for decoy TRAIL-Rs. Furthermore, to achieve specific targeting of selected tumor or stromal cells, bi-functional TRAIL-R1 or -R2 agonistic antibodies or TRAIL fusion proteins have been developed containing a cell-specific binding moiety in addition to a TRAIL-R binding part. For example, a bi-specific melanoma-associated chondroitin sulfate proteoglycan (MCSP)—DR5 (TRAIL-R2) antibody has been produced that combines high affinity binding to melanoma cells with strong apoptosis-inducing potential (126). Similarly, bi-specific antibodies have been developed that allow targeted delivery of TRAIL to surface antigens of T cells to enhance their tumoricidal activity. Moreover, bi-specific antibodies combining PD-L1 immune checkpoint inhibition with TRAIL-induced cell death could counteract an immune suppressive TME and augment T cell activation (127). Notably, the small molecule ONC201, currently evaluated in clinical studies, targets multiple pathways in tumor cells and includes upregulation of *TRAIL* and *TRAIL-R2* transcription. Within tumors ONC201 prompted activation and accumulation of T-cells (CD3^+^, CD4^+^, and CD8^+^) and NK cells thereby selectively potentiating their antitumor activity that involves the TRAIL/TRAIL-R system (128).

**Figure 2:**
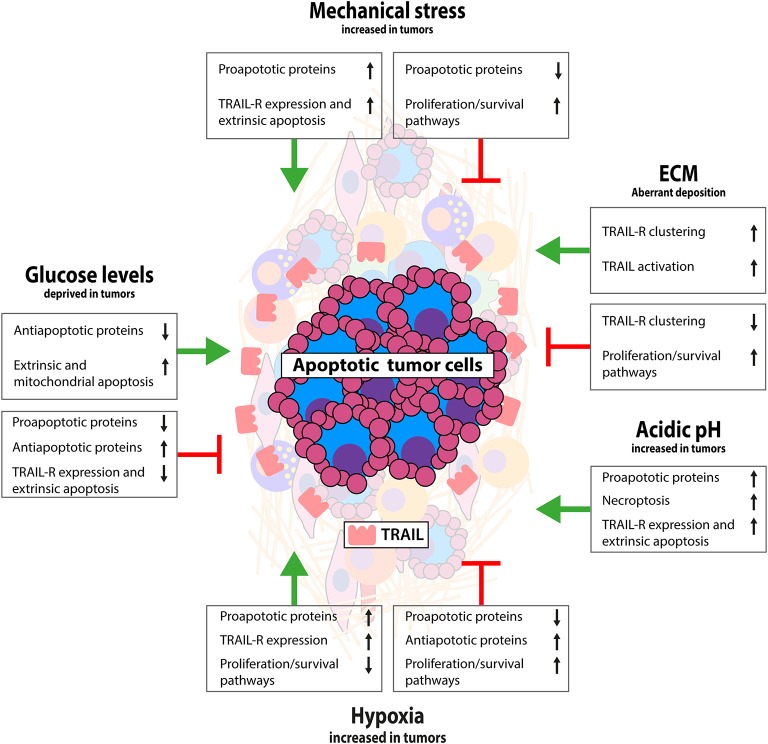
Interactions between tumor and non-cellular TME that modulate TRAIL signaling. Schematic representation of the impact of tumor specific biochemical and biophysical conditions on TRAIL/TRAIL-R signaling. Limited data available thus far indicate mostly a TRAIL sensitizing effect for mechanical stress and acidic pH, resistance by hypoxia and both sensitizing and resistance by ECM and low glucose. Interaction of tumor cells with ECM activates antiapoptotic signaling via integrin signaling, although conversely loss of E-cadherin has been linked with TRAIL resistance. Mechanical stress as a result of external pressure can sensitize for apoptosis and exogenous TRAIL in apoptosis sensitive tumors can reduce interstitial fluid pressure having favorable antitumor effects. Hypoxia and low pH are mostly associated with apoptosis resistance by stimulating antiapoptotic pathways and suppressing mitochondrial apoptosis. Glucose deprivation has been linked with both TRAIL sensitization and resistance, likely depending on chronic or temporal glucose deprived conditions. See text for more details.

The impact of the non-cellular TME on TRAIL signaling in cancer cells is less well-studied. Thus far, studies demonstrated either TRAIL-dependent tumor suppressive or enhancing effects of mechanical stress, hypoxia, acidic pH, and glucose shortage, whereas effects on stromal cells have not been explored until now (see also Figure 2). To appreciate the importance of signals derived from these TME components and to develop targeted strategies, it is essential to gain more insight in these poorly studied underlying mechanisms.

To conclude, in order to improve clinical benefit of TRAIL-R agonists the impact of various components of the TME need to be delineated using appropriate cancer models, which will guide the development of better therapeutic strategies.

## Author Contributions

MdL interpreted literature, drafted the figures, and wrote the manuscript. SdJ corrected and wrote the manuscript. FK interpreted literature, outlined, and wrote the manuscript.

### Conflict of Interest Statement

The authors declare that the research was conducted in the absence of any commercial or financial relationships that could be construed as a potential conflict of interest.
